# MUC1 induces M2 type macrophage influx during postpartum mammary gland involution and triggers breast cancer

**DOI:** 10.18632/oncotarget.23316

**Published:** 2017-12-15

**Authors:** Yuan Li, Zhi Pang, Xinran Dong, Xiaodong Liao, Huayun Deng, Chunhua Liao, Yahui Liao, Guoqiang Chen, Lei Huang

**Affiliations:** ^1^ Department of Pathophysiology, Key Laboratory of Cell Differentiation and Apoptosis of Chinese Ministry of Education, Shanghai Jiao Tong University School of Medicine, Shanghai, P.R. China; ^2^ Department of Endocrinology, Huadong Hospital Affiliated to Fudan University, Shanghai, P.R. China; ^3^ Key Laboratory of Birth Defects, Children’s Hospital of Fudan University, Shanghai, P.R. China; ^4^ Department of Histoembryology, Genetics and Developmental Biology, Key Laboratory of Reproductive Medicine, Shanghai Jiao Tong University School of Medicine, Shanghai, P.R. China

**Keywords:** MUC1-CD, involution, M2 macrophages, p50, breast cancer

## Abstract

The microenvironment of postpartum mammary gland involution (PMI) has been linked to the increased risk of breast cancer and poor outcome of patients. Nevertheless the mechanism underlying regulates the microenvironment remains largely unknown. MUC1, which is abnormally overexpressed in most breast cancer, is physiologically expressed in PMI. Using MUC1 cytoplasm domain (MUC1-CD) transgenic mice, we reveal that the overexpression of MUC1-CD in mammary epithelial cells increases M2 type macrophage infiltration in PMI. By sustain activating p50, MUC1 upregulates M2 macrophage chemo-attractants and the anti-apoptotic protein Bcl-xL. Because of the tumor promotional microenvironments and reduced apoptosis, MUC1-CD delays PMI process and results in atypical phenotype in multiparous mice mammary. This finding is further supported by the positive association between the expression of MUC1 and p50 in Luminal A and Luminal B subtypes through analyzing breast cancer databases. Taken together, our study demonstrates that MUC1-CD plays an important role in regulating microenvironment of PMI and promoting postpartum mammary tumorigenicity, providing novel prevention and treatment strategies against postpartum breast cancer.

## INTRODUCTION

Postpartum mammary gland involution (PMI) is a physiological process of lactation competent gland which returns to a non-lactating state [1]. PMI involves massive epithelial cell death, tissue remodeling, leukocyte infiltration, and adipocyte repopulation [2, 3]. PMI is generally believed to associate with an increased risk of breast cancer. The breast cancer diagnosis in the postpartum period is proved to be an independent risk factor for poor outcomes [4–6]. In murine models, the involution microenvironment is proved to be sufficient to induce mammary tumor growth, invasion and metastasis [7].

The completion of PMI requires a delicate balance between cell death and survival signals. Of note, many of them have been identified as key factors for breast cancer development or progression. Macrophage plays a prominent role in the development of immune responses. It can produce a great number of cytokines, chemokines, reactive oxygen species, etc [8]. One important feature in PMI is the recruitment of macrophages with M2 characteristics, which is crucial for epithelial cell death and adipocyte repopulation [9]. Given that M2 macrophages share similar cytokine profiles and activities with tumor-associated macrophages (TAM), it contributes to the proinflammatory microenvironment and promotes postpartum breast cancer [8]. In addition, Nuclear factor-κB (NF-κB) pathway has been showed playing roles in regulating inflammation and cell survival during PMI. NF-κB activity is increased as early as the first 2 hours of involution [10, 11]. It promotes survival of mammary epithelial cells at the stage of involution through regulating the expression of anti-apoptotic proteins including Bcl-2 and Bcl-xL [12]. NF-κB also regulates immune and inflammatory responses through elevating a series of proinflammatory cytokines release during PMI [13]. Constitutive NF-κB activation was found in several breast cancer cell lines, contributes to breast cancer invasiveness, metastasis, and drug resistance [14, 15]. In contrast, using non-steroidal anti-inflammatory drugs (NSAIDs) to suppress the inflammatory reactions were reported to be protective against tumor invasion and metastasis in PMI [16]. These studies suggest that both M2 macrophages and NF-κB are involved in the development of postpartum breast cancer. However, the mechanism of how PMI process is regulated remains an open question.

MUC1 is one of the transmembrane mucins, expressed abundantly on the apical surfaces of glandular epithelial cells and some hematopoietic cells. It was initially identified as a human breast tumor antigen because of aberrant expressing in more than 90% of human breast carcinomas and associated with poor prognosis [17, 18]. Studies in cell lines have shown that overexpression of MUC1 induces anchorage-independent growth, tumorigenicity and resistance to stress induced apoptosis [19–21]. Studies based on mouse model have further established the roles of MUC1 in the initiation and invasiveness of breast cancer. Overexpression of human MUC1 in the mouse mammary gland drives tumor formation by potentiating EGF-dependent activation of MAP kinase signaling pathways [22]. On the contrary, Muc1 knockout mice show a reduction in tumorigenic phenotype when crossed onto transgenic mice overexpressing oncogene of Wnt-1 [23] or polyomavirus middle T [24] antigen in the mammary gland. The human MUC1 gene consists of N-terminal (MUC1-N) and C-terminal (MUC1-C) subunits that derive from auto cleavage of a single polypeptide, and the two subunits form a stable non-covalent complex at the cell membrane [25]. The MUC1-N subunit contains variable numbers of highly glycosylated 20-amino-acid tandem repeats, it participates in the mucous barrier and supports epithelial growth and survival [22, 26]. The MUC1-C subunit that anchors MUC1-N to the cell surface is composed of a 58-amino-acid extracellular domain, a 28-amino-acid transmembrane domain, and a 72-amino-acid cytoplasmic domain. MUC1-C cytoplasmic domain (MUC1-CD) contains sites for multiple protein interactions, such as receptor tyrosine kinases, and promotes their activation and downstream signals [18]. Our previous studies demonstrate that MUC1-CD is sufficient for inducing tumorigenicity in cells [19]. Overexpression of MUC1-CD in luminal epithelial cells of the mice mammary gland induced an alveolar hyperplasia and hyper-branching phenotype [27]. But the role of MUC1-CD in microenvironment of PMI and the molecular pathways implicated are to be unraveled.

In current study, we found that MUC1-CD induced a sustained activation of p50 and M2 type macrophage infiltration in postpartum involution of transgenic mouse mammary glands, regulated the microenvironment of PMI and triggered hyperplasia and tumorigenesis in multiparous mammary glands.

## RESULTS

### Overexpression of MUC1-CD delays PMI and induces M2 type macrophage influx

**Figure 1:**
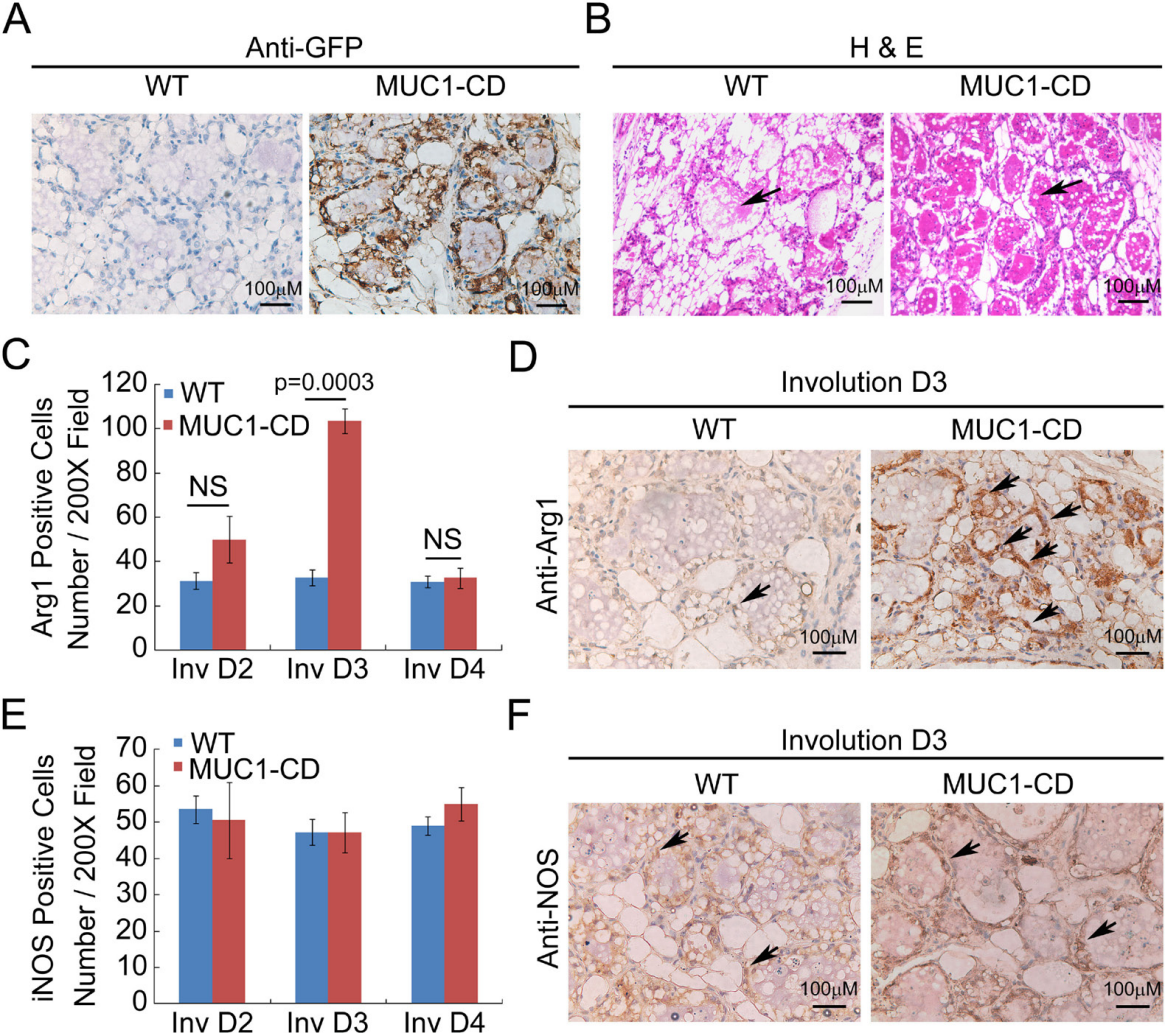
Overexpression of MUC1-CD delays postpartum mammary gland involution and induces M2 type macrophage influx IHC staining of GFP (**A**) and H&E staining (**B**) performed on paraffin sections of wildtype (left panel) and transgenic mouse mammary glands (right panel) at day 3 of involution. Transgenic mammary glands showed delayed alveolar regression (indicated by the arrow). Quantification of the Arginase-1 (Arg 1) (**C**) and iNOS (**E**) positive cells in transgenic and wildtype mice mammary glands at involution day 2,3,4 by IHC staining. For each sample was calculated in 10 high-power fields (*n* = 3 mice per genotype analyzed). Representative images of Arginase-1 (**D**) and iNOS (**F**) IHC staining at day 3 of involution. 400×, scale bars, 100 µm.

To determine whether MUC1-CD effects on postpartum mammary gland involution (PMI), we performed hematoxylin and eosin (H&E) staining of the mammary glands from MUC1-CD transgenic mice and wildtype littermates at day 3 of involution. Notable differences were observed that the mammary glands of transgenic mice were filled with lobuloalveolar structures and engorged with milk; in contrast, the acinar structures of wildtype mammary gland were collapsed and filled with less milk (Figure 1A and 1B). Given that M2 macrophage is crucial for epithelial cell death and adipocyte repopulation during mammary gland involution [9], we wonder if MUC1-CD has an effect on influx of M2 macrophages in PMI. As reported that iNOS expression is used to identify rodent M1 subtype, whereas arginase-1 is used to identify the M2 subtype [28]. Therefore, we analyzed the macrophage subtypes in MUC1-CD transgenic and wildtype mice mammary glands at different stages of involution by IHC staining and quantification. The results showed that the expression of M2 macrophage marker arginase-1 was much higher in MUC1-CD transgenic mice than that in wildtype littermates at the early stage of involution. Peak levels of arginase-1 expression were observed at involution day 3. Whereas the M1 macrophage marker iNOS expression has little, if any, difference between MUC1-CD transgenic and wildtype mice mammary glands (Figure 1C–1F). These data indicate that overexpression of MUC1-CD induces infiltration of M2 macrophage in PMI and delayed PMI process.

### Overexpression of MUC1-CD induces M2-related proinflammatory cytokines

**Figure 2:**
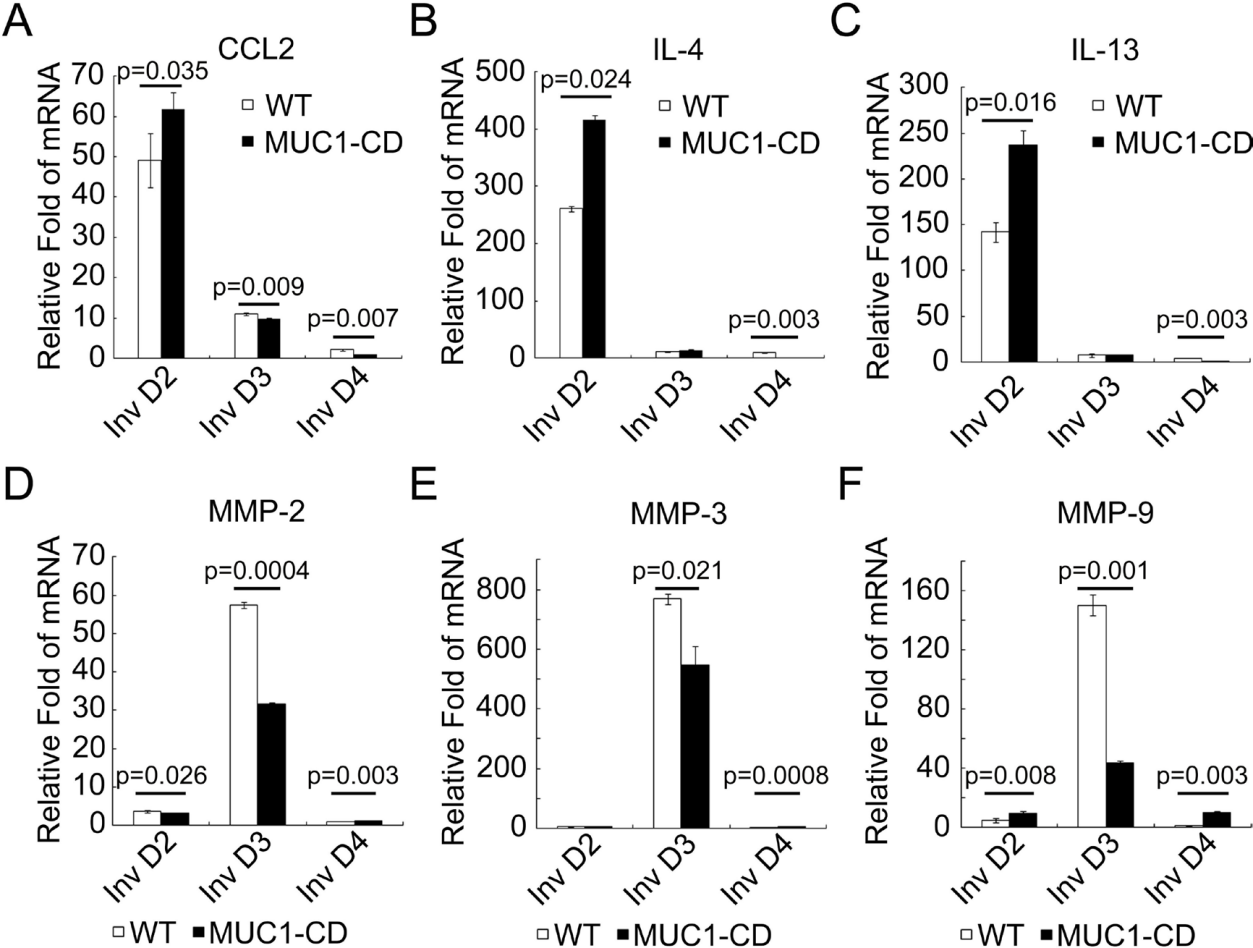
Overexpression of MUC1-CD induces M2-related proinflammatory cytokines (**A**–**F**) Quantitative RT-PCR analysis of M2 associated cytokines (CCL2, LI-4, IL-13) and MMPs (MMP2, MMP3, MMP9) mRNA levels in mouse mammary glands at involution day 2, 3, 4. Results are shown as mean ± S.D. *N* = 3 for each genotype at each time point.

To figure out the mechanism how MUC1-CD induces accumulation of M2 macrophages in PMI mammary glands, we further detected the expression of M2-associated proinflammatory cytokines [28] by quantitative RT-PCR. The mRNA levels of CCL2, IL-4 and IL-13 were notably increased at involution day 2 of MUC1-CD transgenic mice, but decreased at involution day 3 and day 4 in transgenic mice mammary glands. Matrix metalloproteinases (MMPs) are important mediators in cell invasion and immune cell recruitment [29]. The data showed that the mRNA levels of MMP2, MMP3 and MMP9 in transgenic mice mammary glands were considerably decreased at involution day 3, while statistically increased at involution day 4 (Figure 2A–2F). Altogether, these data suggest that overexpression of MUC1-CD accumulates M2 type macrophages by stimulating the expression of M2 macrophage chemo-attractants.

### Overexpression of MUC1-CD sustained upregulates p50 level

**Figure 3:**
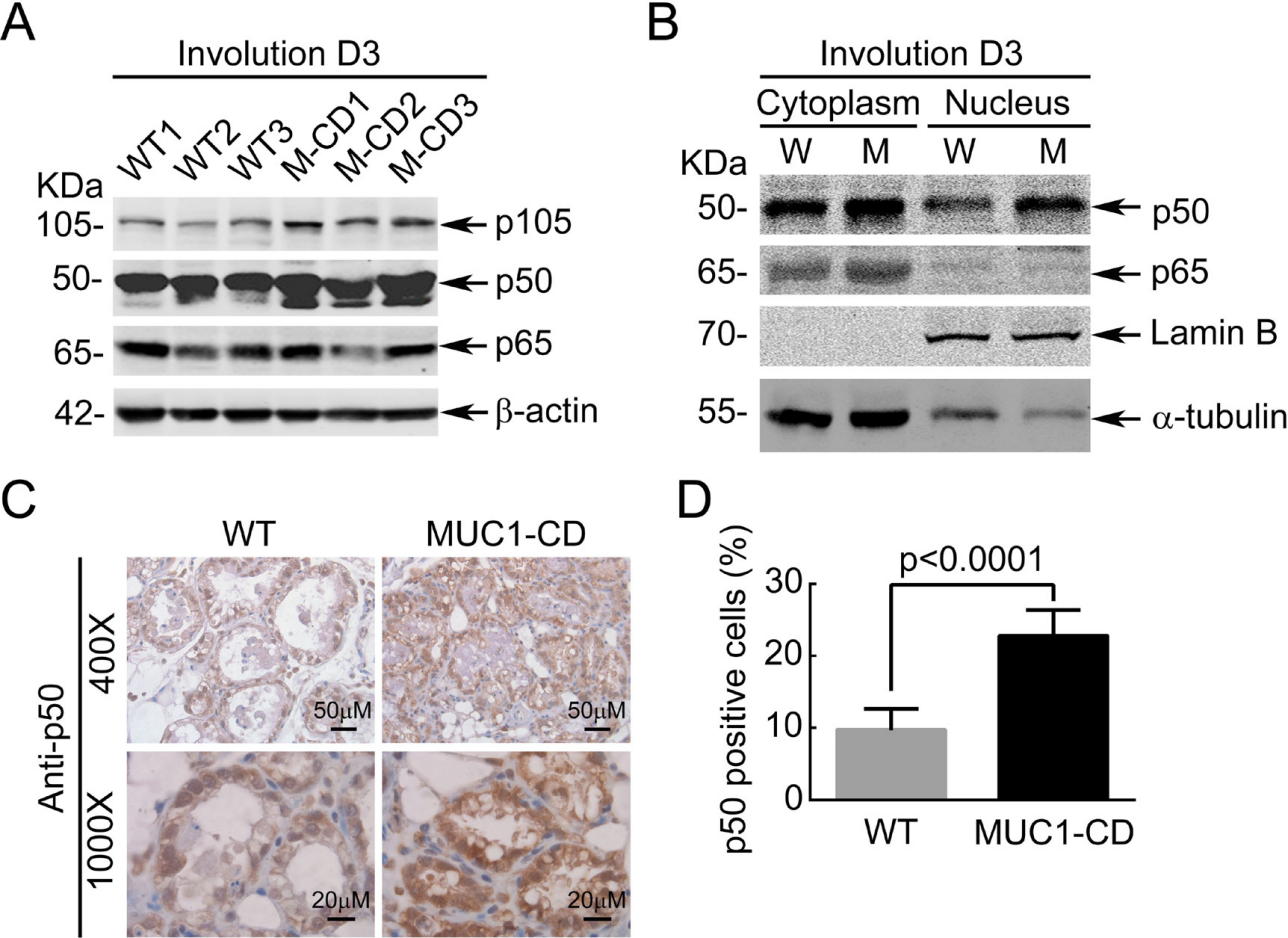
Overexpression of MUC1-CD sustained upregulates p50 level (**A**) Western Blot analysis of p50, p65 in MUC1-CD transgenic and wildtype mice mammary glands at day 3 of involution. (**B**) Nuclear and cytoplasmic fractions of transgenic and wildtype mice mammary glands from day 3 of involution were detected with antibodies against p50, p65, lamin B and α-tubulin. (**C**) Representative images of p50 localization in mammary gland from day 3 of involution by immunohistochemistry. 4 mm sections of paraffin-embedded. 400 ×, bars Indicates 50 μm. 1000 ×, bars indicates 20 μm. (**D**) Quantification analysis of p50 nucleus accumulation in WT and MUC1-CD transgenic mice. Eight separate visual field of each genotype mice were used for statistical analysis (1000×).

Most of M2-associated proinflammatory cytokines are classically transcriptional targets of NF-κB signaling [30–33]. *In vitro* study demonstrated that MUC1-CD activates the Ikappa B kinase beta complex and constitutive NF-κB signaling [34]. Thus, we hypothesize that MUC1-CD may induce the M2-associated proinflammatory cytokines through activating NF-κB signaling. To this end, we detected the protein expression levels of p50 and p65 at involution day 3 in MUC1-CD transgenic and wildtype mice mammary glands. The results showed a markedly increase of p50 expression in MUC1-CD transgenic mice compared to wildtype littermates (Figure 3A). Nuclear and cytoplasmic fractions confirmed that p50 level was noticeably increased in both the nucleus and cytoplasm in MUC1-CD transgenic mice at day 3 of involution (Figure 3B). Consistent with this result, IHC staining of p50 displayed that p50 was located predominantly in the nucleus at involution day 3 in MUC1-CD transgenic mice. While in wildtype mice, the level of p50 in nucleus was lower than that of transgenic mice (Figure 3C and 3D).

### Overexpression of MUC1-CD induces Bcl-xL expression and diminishes apoptosis

**Figure 4:**
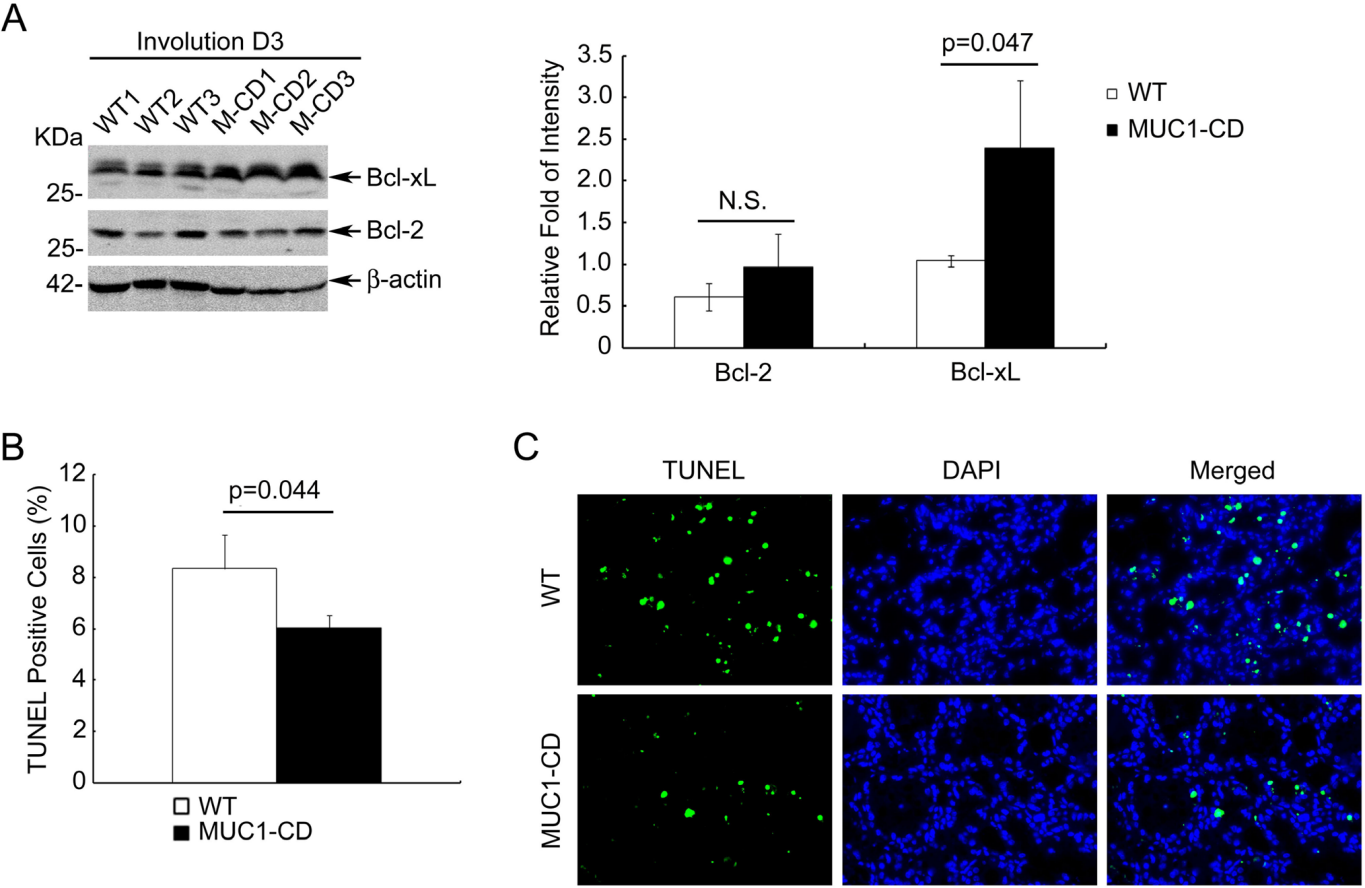
Overexpression of MUC1-CD induces Bcl-xL expression and diminishes apoptosis (**A**) The expression of Bcl-2, Bcl-xL in transgenic and wildtype mice mammary glands at day 3 of involution (left panel). Average protein amounts of Bcl-2 and Bcl-xL from Western blot in left panel were quantified and normalized to the β-actin control. N.S., no significant. *N* = 3 mice per group (right panel). (**B**) Quantification of the apoptotic cells in mammary luminal at day 3 of involution. For each sample was calculated in 10 high-power fields (400×). *N* = 3 mice per genotype analyzed. (**C**) Representative images of TUNEL assays at day 3 of involution. Photomicrographs obtained at a 400× magnification.

In rodents, mammary involution has been characterized that large amounts of the secretary epithelium were eliminated by apoptosis within the first week of involution [35]. To further elucidate the activity of NF-κB, we examined the expression of two anti-apoptotic proteins: Bcl-2 and Bcl-xL these are important transcriptional targets of NF-κB pathway. Consistent with the increased activity of p50, MUC1-CD transgenic mice presented manifestly increased protein levels of Bcl-xL in mammary tissues at day 3 of involution (Figure 4A left and right). In order to define whether MUC1-CD overexpression was associated with reduced apoptosis, we performed TUNEL assays to quantitate the number of apoptotic epithelial cells at involution day 3. Quantitative analysis indicated that there were statistically significant fewer apoptotic cells in the mammary gland luminal of MUC1-CD transgenic mice than that in wildtype mice littermates at involution day 3 (6% versus 8%, *P* = 0.045) (Figure 4B). The representative pictures were shown in Figure 4C. These data demonstrate that overexpression of MUC1-CD diminishes apoptosis and delays postpartum breast involution.

### Overexpression of MUC1-CD prompts hyperplasia and tumorigenesis in multiparous mammary glands

**Figure 5:**
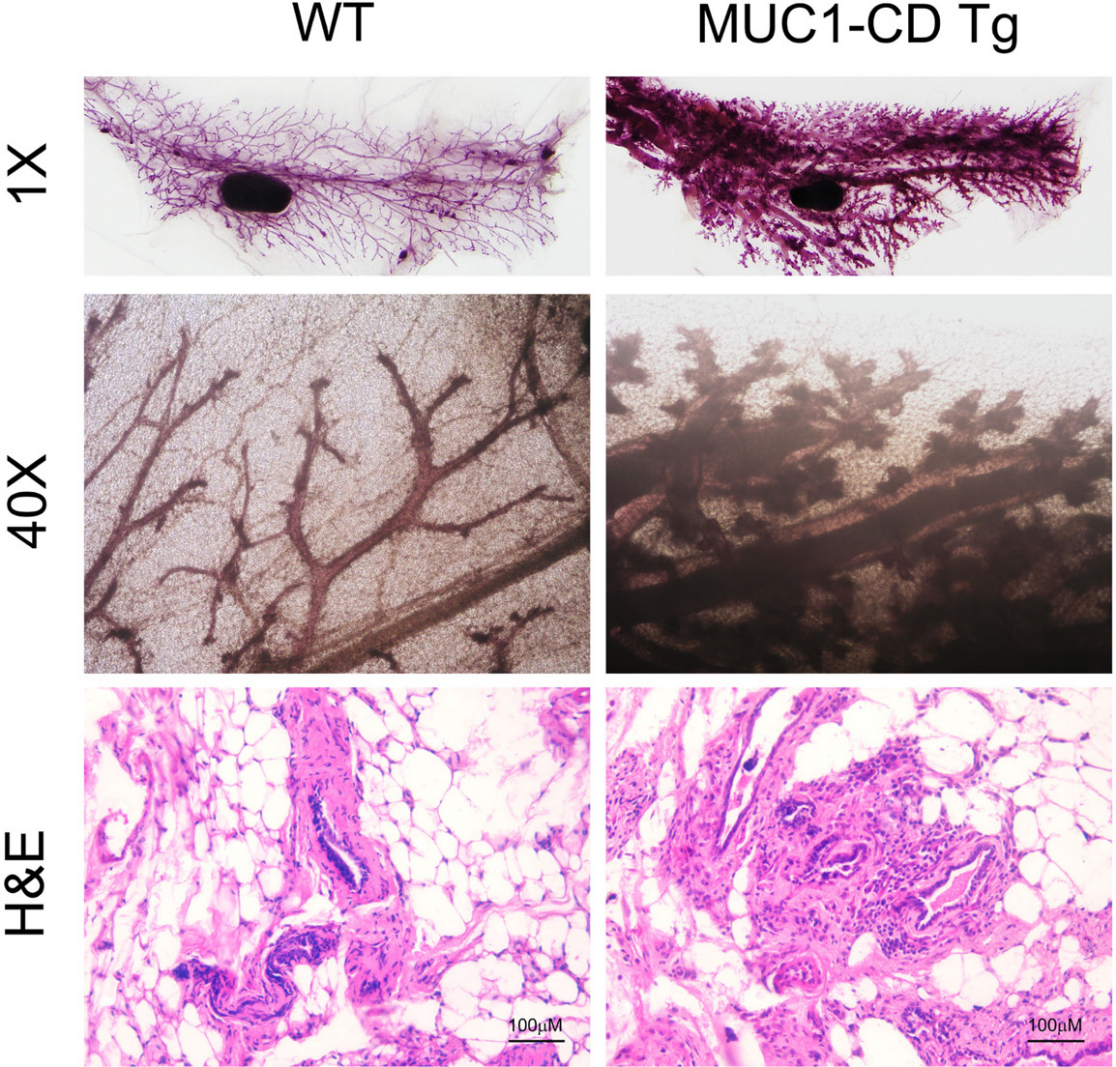
Overexpression of MUC1-CD induces hyperplasia and tumorigenesis in multiparous mammary glands Whole mounts staining (upper and middle) from mammary glands of wildtype (left) and transgenic (right) mice at 20 months of age. Transgenic mammary glands showed more branching and lobuloalveolar structures. Representative H&E images at 20 months of age (lower). Magnification 400×, scale bars, 100 µm.

To further determine whether the function of MUC1-CD in PMI is associated with tumorigenicity in mammary tissue, we analyzed the whole mount mammary gland outgrowths of older multiparous female MUC1-CD transgenic mice and wildtype littermates. Each mouse was allowed to give birth three times at least, and then to be euthanasia by 20 months of age. Whole mount analyses of MUC1-CD transgenic mice showed a dramatic complexity of branching and lobuloalveolar structure compared to those of wildtype mice (Figure 5 upper). To further investigate the detail of mammary ducts, H&E staining of MUC1-CD transgenic mice showed a hyperbranch and histologically atypical phenotype. Some secretory droplets were found in the mammary gland lumen of transgenic mice rather than in wildtype littermates (Figure 5 lower). These data demonstrate that expression of MUC1-CD induces hyperplasia and tumorigenesis in multiparous mammary glands.

### Overexpression of MUC1-CD provokes M2 type macrophage influx in multiparous transgenic mice mammary glands

**Figure 6:**
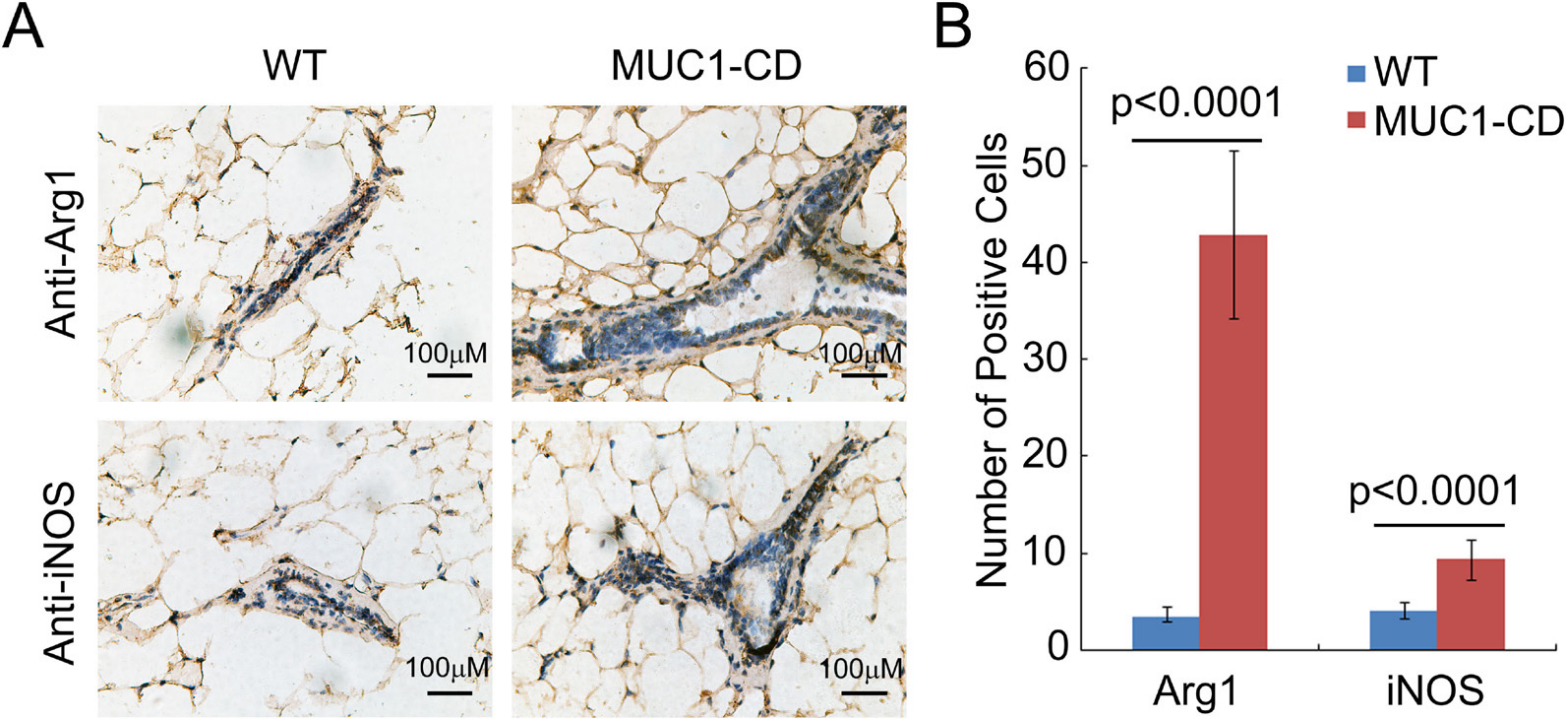
Overexpression of MUC1-CD provokes M2 type macrophage influx in multiparous transgenic mice mammary glands (**A**) Representative images of iNOS and Arginase-1 IHC staining from multiparous MUC1–CD transgenic mice and wildtype controls at 20 months of age. Magnification 400×, scale bars, 100 µm. (**B**) Quantification of the iNOS and Arginase-1 positive cells. For each sample was calculated in 10 high-power fields (400×). *N* = 3 mice per genotype analyzed.

To further explore the relationship between MUC1-CD induced phenotype and M2 type macrophage activity in older multiparous mice, we conducted IHC and quantification analysis of arginase-1 and iNOS in multiparous MUC1-CD transgenic and wildtype mice mammary tissues at 20 months of age. Consistent with the results of involution, the expression of arginase-1 was much higher in MUC1-CD transgenic mice (right) than that in wildtype littermates (left). Although the expression of iNOS was significantly higher in transgenic mice than that in wildtype mice, it was weak in both transgenic and wildtype mice mammary epithelium cells (Figure 6A and 6B). These data confirm that overexpression of MUC1-CD triggers hyperplasia and tumorigenesis by inducing infiltration of M2 macrophage in multiparous mammary glands.

### Positive correlation between MUC1 and p50 in breast cancers

**Figure 7:**
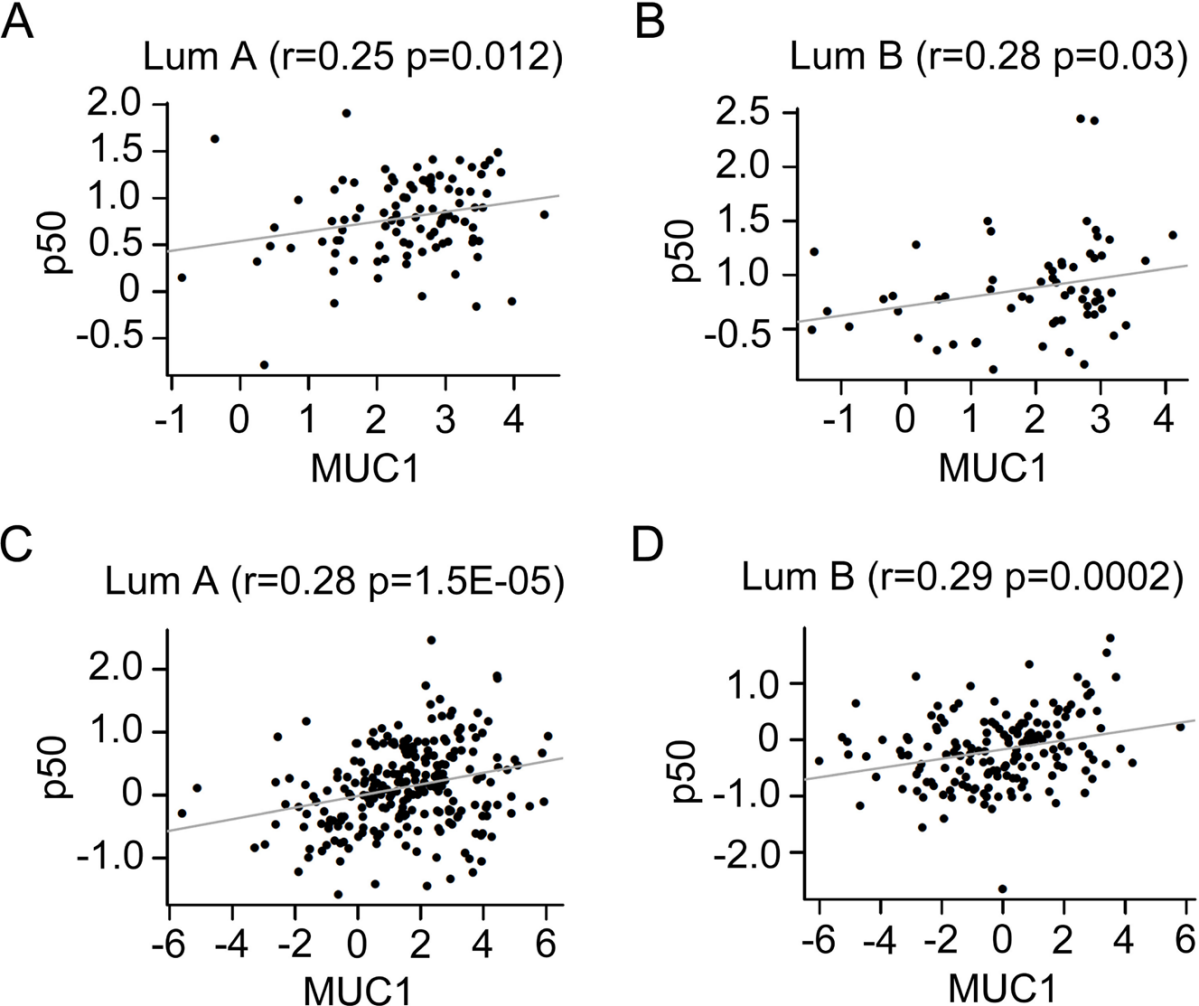
Positive correlation between MUC1 and p50 in different subtype breast cancers Relationships between the expression of MUC1 and p50 in Luminal A (**A**), Luminal B (**B**) subtype in GSE18229 dataset (*N* = 377). Relationship between MUC1 levels and the expression of p50 in Luminal A (**C**), Luminal B (**D**) subtype in combined 855 datasets.

To investigate the association of MUC1 with p50 in human breast cancers, we collected two independent large datasets for co-expression analysis between MUC1 and p50. Both two datasets are annotated five subtypes of human breast cancer. Spearman correlation analysis indicated a positive association between the expression of MUC1 and p50 in Luminal A (*r* = 0.25, *P* = 0.012) and Luminal B (*r* = 0.28, *P* = 0.03) subtype breast cancer in GSE1822 dataset (Figure 7A and 7B). In combined 855 datasets, MUC1 was positively associated with p50 in Luminal A (*r* = 0.28, *P* = 1.5E-05) and Luminal B (*r* = 0.29, *P* = 0.00015) subtype (Figure 7C and 7D). These data suggest a possible mechanism of MUC1 in breast cancer initiation through activating p50 and thereby regulating the tumor-related environment.

## DISCUSSION

To illuminate the mechanism of how the microenvironment of postpartum mammary gland involution (PMI) is established will explore immunotherapeutic targets for postpartum breast cancer. Our study investigated the effect of MUC1-CD in PMI stage at which MUC1 is physiologically activated [36]. We found that the overexpression of MUC1-CD in mice mammary epithelium cells activates p50 and following up-regulates anti-apoptotic Bcl-xL as well as M2-associated pro-inflammatory cytokines. In line with these, MUC1-CD delays the process of PMI and alters the microenvironment by recruiting M2 macrophages. The inflammation microenvironment in PMI further induced hyperplasia and atypical phenotype in multiparous transgenic mice. Our data discovered a novel insight into postpartum breast cancer that brings important therapeutic significance.

Previous studies reported that transgenic mice overexpressing full-length human MUC1 rather than MUC1ΔCT (human MUC1-CD deletion) exhibited delayed postlactational involution and tumorigenesis [37], indicating a potential role of MUC1-CD in PMI. We provide a direct *in vivo* evidence that overexpression MUC1-CD in transgenic mice triggers delayed postlactational involution and histologically atypical phenotype, supporting the oncogenic function of this subunit. The relationship between inflammation microenvironment and postpartum breast cancer has received a great deal of attention recently [16, 38, 39]. Among the inflammation microenvironment, macrophages were found to be essential for execution of cell death during involution. Macrophages are divided into the M1/M2 categories. M1 macrophages are involved in antigen presentation, immune surveillance, and killing of cells with foreign antigens, including tumor cells. M2 macrophages participate in tissue repairing through activities including phagocytic debris clearance. As we mentioned previously, M2 macrophages have similar cytokine profiles and functional phenotypes to tumor-associated macrophages (TAMs) which modulate the tumor microenvironment, enhancing metastatic potential and resistance to treatments [8, 40, 41]. An *in vitro* study reported that decreased MUC1 protein expression accompanied by decreased M2-TAM markers CD68^+^/CD163^high^ whereas M1 markers CD68^+^/CD80^high^ increased, suggesting that MUC1 may be involved in promoting M2-TAM polarization [42]. Consistent with previous reports, MUC1-CD significantly increased accumulation of M2 macrophages in weaning and multiparous mammary tissues, suggested that MUC1-CD could initiate tumorigenesis via recruiting M2 macrophages. Of note, recent studies demonstrated that macrophages isolated from human breast tumors could release epithelial growth factor (EGF) [43], while MUC1 has been reported to enhance EGFR expression and involving in neoplasia and cell adhesion [22]. These may further promote the development of tumors.

During the stage of PMI, a series of immune-related genes expression were increased in the absence of inflammatory insult, such as interleukins (ILs), CC-chemokine ligand 2 (CCL2) and MMPs, which are also related to breast cancer development [13]. Th2 cytokines IL4 and IL13 which could recruit M2-type macrophages in the immune system [8], have been proved to be involved in epithelial cancer cells survival, proliferation and migration [44, 45]. CCL2 is a highly potent chemo-attractant for monocytes and macrophages to sites of tissue injury and inflammation [30]. It is overexpressed in different cancers [46] and is associated with poor prognosis in breast cancer [47]. Studies in mice have implicated that overexpression of CCL2 increases recruitment of macrophages to the mammary gland and increases susceptibility to DMBA induced mammary gland cancer [48]. MUC1-CD transgenic mice display manifestly increased mRNA levels of IL4, IL13 and CCL2 in their mammary tissues at involution day 2, suggest that recruitment of M2 macrophages by MUC1-CD likely occur through upregulation of M2 macrophage chemo-attractants. MMPs are important in tissue remodeling [49]. Increased expression of MMPs could permit the lactation competent mammary gland regressing to a non-lactating state. MMP2, MMP3 knock-out mice displayed alterations to mammary gland structure and the impairment of lactation [50, 51]. Both MMP2 and MMP9 are known to cleave collagen I [52], which has been proved to be highly chemotactic for immune cells [1]. In the present study, we observed that the expression levels of MMP2, MMP3 and MMP9 were decreased in transgenic mice mammary glands at involution day 3. This may also have contributed to the delayed postpartum involution phenotype that we observed in MUC1-CD transgenic mice aside of apoptosis reduction.

NF-κB is considered to be the major link between inflammation and tumorigenesis. A number of studies have revealed dysregulation of NF-κB could lead to the constitutive overexpression of pro-inflammatory cytokines [53]. There are five NF-κB family members in mammals: RelA (p65), RelB, c-Rel, NF-κB1 (p50 and its precursor p105) and NF-κB2 (p52 and its precursor p100) [38]. In the canonical NF-κB pathway, NF-κB dimers (composed of the p65 and p50 subunits) are held inactive in the cytoplasm through binding of IκB proteins. The stimuli activate the IκB kinase complex, leading to phosphorylation, ubiquitination and degradation of IκB proteins and release NF-κB dimers translocate to the nucleus, bind specific DNA sequences and promote transcription of target genes such as anti-apoptotic protein Bcl-xL [54, 55]. It was previously reported that MUC1-C binds directly to p65 and blocks the interaction between p65 and its inhibitor IκBα, contributes to NF-κB-mediated transcriptional activation in carcinoma cells [56]. Our data shows an elevated p50 level rather than p65 level in the nucleus in MUC1-CD transgenic mice. The mechanism of how MUC1 affects different dimmers *in vivo* and *in vitro* remains unknown. It may depend on cell type, complexity environment *in vivo* and the effects of weaning hormones. In line with our observations, recent research found that p50-deficient mice showed exacerbated M1-driven inflammation and defective M2-polarized inflammatory reaction, associated with tumor growth reduction and survival prolongation [57]. This further suggests that the activation of NF-κB pathway is a key component in the orchestration of M2-driven inflammatory reactions and upregulation of Bcl-xL expression in MUC1-CD transgenic mice mammary glands.

Coincident with previous data, we also observed that MUC1-CD increased M2 type macrophage influx, induced hyperbranch and histologically atypical phenotype in older multiparous transgenic mice. This suggests that the complexity immune microenvironment of the involution gland in MUC1-CD transgenic mice may be associated with tumor progression.

In support of our views, recent expression data analysis indicated that the expression of MUC1 is up-regulated in Pregnancy Associated Breast Cancer (PABC) tissues [58]. Nowadays, genomic studies have defined five main breast cancer subtypes (Luminal A, Luminal B, HER2-enriched, basal-like, claudin-low), each of them shows significant differences in incidence, survival and response to therapy [59, 60]. To confirm our finding, we chose 2 large breast cancer databases to analyze the expression of MUC1 and p50 in different subtypes. Results showed that MUC1 was positively associated with p50 in Luminal A and Luminal B subtypes across two independent breast cancer data sets. Approximately 90%–95% ER-positive/HER2-negative breast cancers belong to Luminal A and B subtypes [61]. Compared to Luminal A tumors, Luminal B subtype shows higher expression of proliferation genes and worse baseline distant recurrence-free survival at 5- and 10-years [62]. Both luminal subtypes have shown to derive a relative benefit from endocrine therapy, but several luminal tumors harbor resistance to endocrine therapy [63]. Previous studies have shown that MUC1-CD contributes to the regulation of genes that are highly predictive of clinical outcome in breast patients [64] and predicts failure to tamoxifen treatment [65]. We hypothesize that overexpression of MUC1 and p50 may be one of the mechanisms for endocrine resistance.

In summary, our data provide *in vivo* evidence for the function of MUC1-CD in the PMI and postpartum breast cancer, suggests that MUC1-CD-targeted therapies toward the window of PMI of corresponding subtypes could be preventive for postpartum breast cancers.

## MATERIALS AND METHODS

### Mice and procedures

All animal experiments were conducted in accordance with the ARRIVE guidelines [66]. Research was approved by the Institutional Animal Care and Use Committee at the Shanghai Jiaotong University School of Medicine (Approval ID: A-2015-001). All mice were from the C57BL/6J strain. They were housed under similar conditions, and a similar fraction. The MUC1-CD transgenic mice were generated as described [27]. First-pregnancy mice were analyzed. Excess pups were removed after birth to make the experimental mice breed 5–6 pups. They were allowed to lactate for 8–10 days, and the day when the pups were removed was counted as day 0 of involution. Mammary glands were harvested at 2-, 3-, 4-, and 7-day time points after involution.

### Whole-mount staining

For whole-mount staining, the inguinal mammary glands were spread on glass slides, fixed overnight in Carnoy’s fixative (60% ethanol, 30% chloroform, 10% glacial acetic acid) at room temperature, rehydrated and stained in carmine alum overnight. Then the slides were washed in 70%, 95%, 100% ethanol gradually for 15 min and cleared in xylene. Digital images were acquired with Olympus digital camera.

### Histological and immunohistochemical analysis

Mammary tissues were fixed in 4% paraformaldehyde in PBS at room temperature for 18 to 24 hours, dehydrated in ethanols, embedded in paraffin. For histological analysis, 6 µm sections were cut and stained with Hematoxylin and Eosin. For immunohistochemical analysis, slides were deparaffinized with xylene, dehydrated in decreasing concentrations of ethanol, and then processed in 10 mM citrate buffer (pH 6.0) and heated to 92–96°C for 30 min for antigen retrieval. Tissue sections were treated with 3% hydrogen peroxidase in PBS for 10min to block endogenous peroxidase activity. After blocking for 1h in 0.5% goat serum, the sections were incubated with primary antibodies overnight at 4°C. The staining procedure was followed the manufacturer’s instructions of ABC staining system (Santa-Cruz Biotechnology, Santa Cruz, CA, USA). Anti-MUC1-C antibody (Thermo Scientific, Hudson, NH, USA), anti-p50 (Stressgen, San Diego, CA, USA), anti-p65 (Neomarkers, Fremont, CA, USA), anti-Bcl-xL (Santa-Cruz Biotechnology, CA, USA) antibodies were used, respectively, for different section staining. The intensity quantified by Image J software.

### TUNEL assay

To detect apoptotic nuclei, paraformaldehyde-fixed the number four mammary glands (at day3 of involution) were analysed. Paraffin sections were analyzed by DeadEnd™ Fluorometric TUNEL System (Promega, Madison, WI, USA) following manufacturer’s instructions. For each sample was calculated in 10 high-power fields (400×) (a minimum of 2000 cells per sample were counted). The number of apoptotic cells was calculated as a percentage of total cell count. The averages of three to four mice per genotype were determined.

### Quantitative real-time PCR (RT-qPCR)

Total RNA was extracted from third mammary glands with Trizol reagent (Invitrogen, Carlsbad, CA, USA) according to manufacturer’s instructions. 2 ug of total RNA was treated with RNase-free DNase I (Promega, Madison, WI, USA) for 10 minutes at 37°C to remove any contaminating DNA, and reverse transcribed with AMV reverse transcriptase (20U; Takara, Otsu, Japan). Quantitative RT-PCR was performed with SYBR Green PCR kit (Takara, Otsu, Japan) using ABI PRISM 7500 Sequence Detection System (Applied Biosystems, CA, USA) according to the manufacturer’s instructions. The results were normalized to β-actin expression and evaluated using the ΔΔCt relative quantification method. The following primers were used:

**Table:** 

mIL-4-F	5′ GGTCTCAACCCCCAGCTAGT 3′
mIL-4-R	5′ GCCGATGATCTCTCTCAAGTGAT 3′
mIL-13-F	5′ CCTGGCTCTTGCTTGCCTT 3′
mIL-13-R	5′ GGTCTTGTGTGATGTTGCTCA 3′
mCCL2-F	5′ TTAAAAACCTGGATCGGAACCAA 3′
mCCL2-R	5′ GCATTAGCTTCAGATTTACGGGT 3′
mMMP2-F	5′ CAAGTTCCCCGGCGATGTC 3′
mMMP2-R	5′ TTCTGGTCAAGGTCACCTGTC 3′
mMMP3-F	5′ ACATGGAGACTTTGTCCCTTTTG 3′
mMMP3-R	5′ TTGGCTGAGTGGTAGAGTCCC 3′
mMMP9-F	5′ CTGGACAGCCAGACACTAAAG 3′
mMMP9-R	5′ CTCGCGGCAAGTCTTCAGAG 3′
β-actin-F	5′ GTGGGAATGGGTCAGAAGGA 3′
β-actin-R	5′ CTTCTCCATGTCGTCCCAGT 3′

### Western blot analysis

Protocols for immunoblotting analysis have been described in detail previously [19]. Tissue lysates were prepared from the third mammary glands. Equal amounts of protein were separated by SDS-PAGE and transferred to nitrocellulose membrane. The membrane was probed with the primary antibody overnight at 4°C, and incubated with secondary antibodies conjugated with the HRP for 30min. Finally, the membrane was washed and scanned with an Odyssey Infrared Imaging System (LI-COR Biotechnology, Lincoln, NE, USA). The primary antibodies used in this study were mouse anti-tubulin(Sigma-Aldrich Co., St. Louis, MO, USA), rabbit anti-p50, rabbit anti-p65, rabbit anti-β-catenin, goat anti-lamin B and goat anti-β-actin (Santa-Cruz Biotechnology, Santa Cruz, CA, USA). Nuclear and cytosolic fractions were prepared by the NE-PER Nuclear and Cytoplasmic Extraction Reagents kit (PIERCE Biotechnology, Rockford, IL, USA) according to the manufacturer’s instructions. The protein levels were quantified by Quantity One software.

### Human breast tumor microarray data sets

For gene expression correlation analysis, two large breast cancer datasets: GSE18229 and combined 855 datasets were collected. The dataset GSE18229 was downloaded from the publicly available genomic data repository NCBI GEO (Gene Expression Omnibus) database (*N* = 337). Combined 855 datasets were obtained from (https://genome.unc.edu/pubsup/breastGEO/855dataset.NKI295.EMC192.EMC286.MSK82.DWD.std.Aug2010.txt) (*N* = 855). The raw datasets were preprocessed using limma package in R (3.3.3). The gene expression profile for each sample was normalized by subtracting the mean expression value and divided by standard deviation. The expression profile of MUC1 across all samples was set as the reference profile. Spearman correlation analysis was used to determine the association between expression levels of MUC1 and p50. The *p*-value threshold was set at 0.05.

### Statistical analysis

The data are presented as the mean ± standard deviation. The differences between the various experimental groups were analyzed by Student’s *t*-test. *P* < 0.05 were deemed as statistically significance.
